# Assessment of Knowledge of Symptoms of Ischemic Heart Disease in Population Visiting a Tertiary Care Hospital in Pakistan

**DOI:** 10.7759/cureus.5482

**Published:** 2019-08-25

**Authors:** Sahibzada M Rasool, Zulqarnain Asad, Awais A Bhatti, Afifa Kulsoom, Noman A Chaudhary, Amina S Rasool, Abdullah Sadiq

**Affiliations:** 1 Internal Medicine, Rawalpindi Medical University, Rawalpindi, PAK; 2 Surgery, Rawalpindi Medical University, Rawalpindi, PAK; 3 Community Medicine, Rawalpindi Medical University, Rawalpindi, PAK

**Keywords:** knowledge, ischemic heart disease, population

## Abstract

Introduction

Cardiovascular diseases are an important cause of mortality in Pakistan. Developing nations like Pakistan with poor literacy rates and the majority of the population living in rural areas seem to be insufficient in their knowledge of symptoms. A study indicated that about half of the cardiac deaths occur within one hour of onset of symptoms, thus it is necessary to have adequate knowledge of symptoms to identify the sufferer and to pursue medical services as early as possible. The aim of our study was to assess the knowledge of ischemic heart disease (IHD) symptoms in the population and to investigate the relationship of age, gender, socio-economic status, education, and occupation with knowledge.

Materials and Methods

This was a descriptive cross-sectional study carried out in the Holy Family Hospital, Rawalpindi, Pakistan over a period of four months from May 2018 to August 2018. The study population comprised of people visiting the hospital. Individuals aged 18 and above were included while medical professionals were excluded. An interviewer-assisted semi-structured questionnaire was used as the data collection tool. After taking consent, 225 participants were asked about their demographic profile and to enlist as many symptoms of IHD as possible. Reference was made to the seven typical symptoms of IHD as recognized by the World Health Organization (WHO). Statistical Package for Social Sciences (SPSS), v23.0 (IBM SPSS Statistics, Armonk, NY) was used for the analysis. Independent samples t-test and one-way ANOVA test were applied; p ≤ 0.05 was considered significant.

Results

Out of the seven symptoms endorsed by WHO, chest pain was most frequently identified (42%), followed by pain in the arm (23%), diaphoresis (19%), weakness and fainting (16%), dyspnea (15%), paleness (8%), and sickness and vomiting (5%). Mean score, out of seven symptoms, was 1.28 ± 1.19. Among the total participants, 34% could not enlist any symptom. Participants with higher education, skilled workers, and those having relatives who suffered from IHD showed significantly higher knowledge about IHD symptoms.

Conclusions

The study showed a paucity of knowledge about IHD symptoms among the participants. Hence it provides grounds for future awareness campaigns to educate the masses.

## Introduction

Cardiovascular diseases (CVD) are recognized as the most common cause of death worldwide. In 2013, an estimated 17 million i.e. 32% of deaths globally, were attributable to cardiovascular diseases [1]. People of Indo-Asian origin have one of the highest susceptibilities to coronary artery disease [2, 3]. In Pakistan, cardiovascular diseases account for 19% of the total deaths [4]. The burden of CVD is constantly rising in developing countries because of the adoption of western lifestyle and urbanization [5, 6]. In Pakistan, the majority of the population is currently living in rural areas (60-65%), however people are migrating to the larger cities, and Pakistan will become a predominantly urban country by the year 2030. The literacy rate of Pakistan varies from 97% (Islamabad) to 20% (District Kohlu) [7]. Developing countries like ours, with poor literacy rates and lack of awareness of symptoms, do not have the resources to cope with this burden [8].

Ischemic heart disease (IHD) is a time-critical illness. A study points out that approximately half of the IHD victims die within one hour of symptom onset before even reaching the hospital [9]. The delay between the onset of symptoms and treatment can occur at different levels of pre-hospital care, including patient delay and transport delay [10]. The lack of knowledge of IHD symptoms is a major cause of patient delay [11]. In order to ensure survival, early access to health care, and the provision of advanced treatment is essential. This requires recognition of warning symptoms of the IHD and calling for emergency services as early as possible [12]. Therefore this study aims at assessing the knowledge of the population regarding the symptoms of IHD and to investigate its relationship with variables like age, gender, socio-economic status, education, and occupation. 

## Materials and methods

This was a descriptive cross-sectional study carried out in the Holy Family Hospital, Rawalpindi, Pakistan from May 2018 to August 2018. The study population comprised of people visiting the hospital. Patients were included using non-probability convenient sampling. All the individuals aged 18 and above were included irrespective of their IHD experience, while medical professionals were excluded. An interviewer-assisted semi-structured questionnaire was used. This study comprised of 225 participants. After taking informed consent, they were asked about the demographic profile, educational status, socioeconomic status, history of ischemic heart disease, any close relative suffering from ischemic heart disease, and to enlist as many symptoms of ischemic heart disease as possible.

Reference was made to the seven typical symptoms of IHD as recognized by World Health Organization (WHO) which include: central chest pain/discomfort, pain in the arm/shoulder/jaw/back, breaking into the cold sweats (diaphoresis), feeling sick or vomiting, weakness/lightheadedness/fainting, shortness of breath, and becoming pale [13]. Hence a participant could have a maximum score of seven while a minimum score of zero. 

Statistical Package for Social Sciences (SPSS), v23.0 (IBM SPSS Statistics, Armonk, NY) was used for analysis. Frequencies, percentages, means and standard deviations were calculated. Tables were constructed comparing each variable with the corresponding mean score. Independent samples t-test test and one-way ANOVA test were applied. Level of significance was taken as p ≤ 0.05. 

## Results

A total of 225 respondents were included in the study. Age ranged from 18 to 85 years, with a mean of 36.49 ± 12.97 years. The sample consisted of 128 (56.9%) males and 97 (43.1%) females. The mean score of knowledge about IHD was 1.28 ± 1.18 out of seven (Table 1).

**Table 1:** Frequency of age and gender with the corresponding mean score SD - standard deviation *p-value ≤ 0.05 was considered as significant

Variables	Categories	Frequency (%)	Mean score ± SD	p-value*
Age	<35yrs	94 (41.8%)	1.18 ± 1.06	0.508
	≥35yrs	131 (58.2%)	1.34 ± 1.28	
Gender	Male	128 (56.9%)	1.21 ± 1.13	0.462
	Female	97 (43.1%)	1.36 ± 1.26	

The sample consisted of 107 (47.6%) unemployed participants, 23 (10.2%) were skilled workers, 63 (28.0%) were semi-skilled workers, 18 (8%) were unskilled, 13 (5.8%) were semi-professional, and one (0.4%) was professional (Table 2).

**Table 2:** Frequency of different occupations and corresponding mean scores SD - standard deviation *p-value ≤ 0.05 was considered as significant

Occupation	Frequency (%)	Mean Score ± SD	p-value*
Professional	1 (0.4%)	2.00 ± 0.00	0.003
Semi-Professional	13 (5.8%)	1.69 ± 1.65	
Skilled	23 (10.2%)	1.87 ± 1.06	
Semi-Skilled	63 (28.0%)	1.04 ± 1.06	
Unskilled	18 (8.0%)	0.56 ± 1.04	
Unemployed	107 (47.6%)	1.34 ± 1.21	

There were 174 (77.3%) participants from the urban areas and 50 (22.7%) participants from the rural areas. Mean score difference between the two groups was statistically insignificant (p > 0.05). One hundred and eighty-one (80.4%) participants were married while 44 (19.6%) were not married.

People with higher education had a better understanding of the symptoms of ischemic heart disease (p < 0.000). The following table shows the distribution of participants based on their educational status and respective mean scores (Table 3).

**Table 3:** Educational status of participants and the corresponding mean score SD - standard deviation *p-value ≤ 0.05 was considered as significant

Educational status	Frequency (%)	Mean Score ± SD	p-value*
None	44 (19.6%)	0.75 ± 1.10	<0.000
Primary (up to grade 5)	23 (10.2%)	1.13 ± 1.42	
Secondary (up to grade 8)	38 (16.8%)	0.86 ± 1.16	
Matric (up to grade 10)	62 (27.6%)	1.52 ± 1.11	
College (up to grade 12)	24 (10.7%)	1.46 ± 1.06	
University	34 (15.1%)	1.94 ± 0.98	

Five (2.2%) participants suffered from IHD while 220 (97.7%) did not. The knowledge score difference between the two groups was found to be insignificant (p > 0.05). Participants whose relatives suffered from IHD were 81 (36.0%) and those who had no relatives ever suffering from IHD were 144 (64.0%). The difference in mean score between these two groups was statistically significant (p < 0.001) (Table 4).

**Table 4:** Relationship of IHD experience with the mean score SD - standard deviation IHD - ischemic heart disease *p-value ≤ 0.05 was considered as significant

Variables	Categories	Frequency (%)	Mean score ± SD	p-value*
IHD (self)	Suffered	5 (2.2%)	2.20 ± 1.48	0.108
	Not Suffered	220 (97.8%)	1.26 ± 1.18	
IHD (relative)	Suffered	81 (36.0%)	1.73 ± 1.14	<0.000
	Not Suffered	144 (64.0%)	1.02 ± 1.14	

The following table shows the distribution of participants based on their socio-economic status and respective mean scores (Table 5).

**Table 5:** Socio-economic status and the corresponding mean score SD - standard deviation PKR - Pakistani Rupee *p-value ≤ 0.05 was considered as significant

Variables	Categories	Frequency (%)	Mean score ± SD	p-value*
Income (PKR)	<10,000/month	31 (13.8%)	1.10 ± 1.49	0.074
	10,000-30,000/month	120 (53.3%)	1.18 ± 1.15	
	30,001-50,000/month	47 (20.8%)	1.38 ± 1.05	
	50,001-70,000/month	13 (5.8%)	1.62 ± 1.12	
	70,001-90,000/month	4 (1.8%)	2.50 ± 1.29	
	>90,000/month	10 (4.4%)	1.60 ± 1.07	

**Figure 1:**
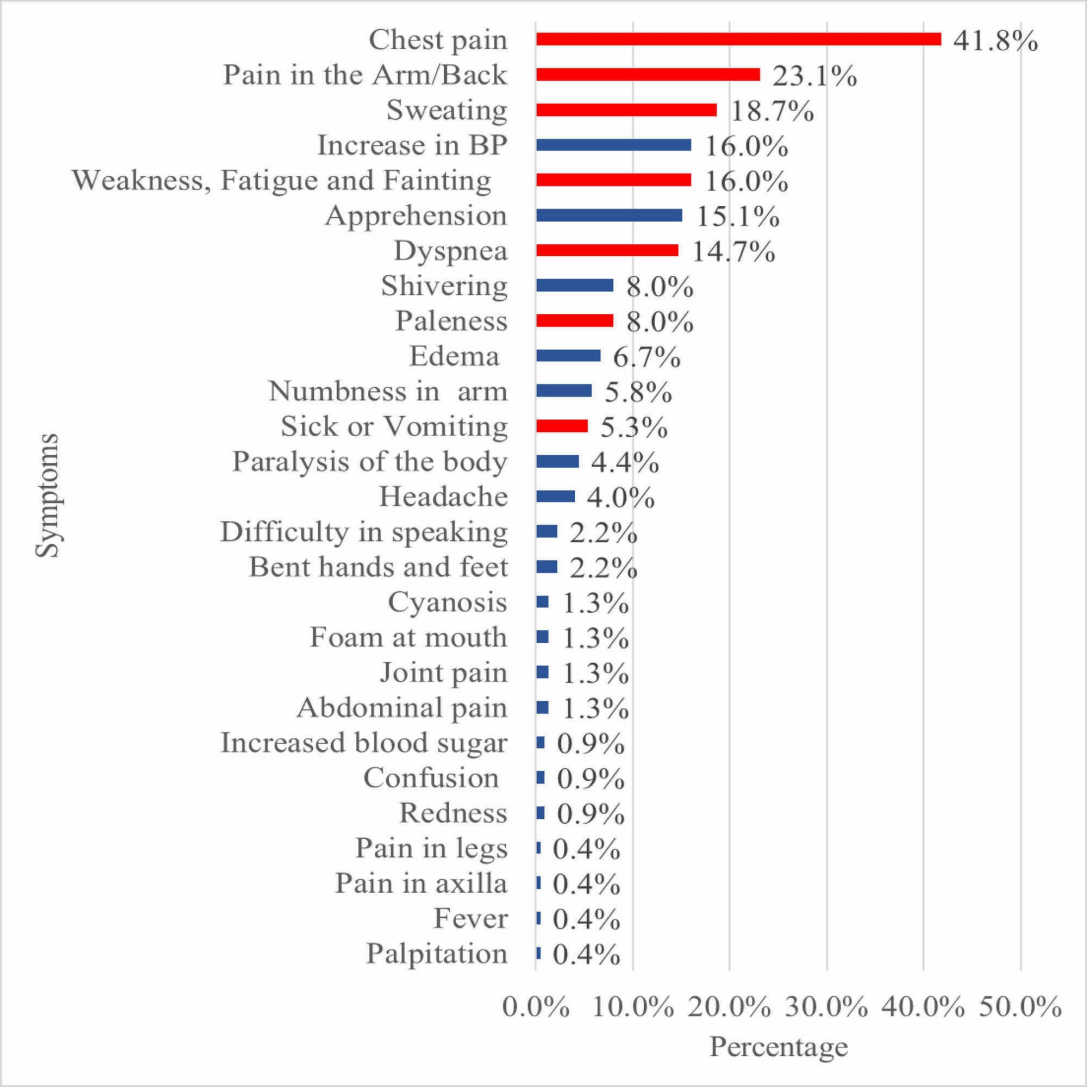
Participants' responses about symptoms of ischemic heart disease

The following figure shows the responses of the participants regarding the symptoms of IHD. A total of 27 different responses were recorded. The red bars show the responses in line with typical symptoms endorsed by WHO, while the blue bars are the additional unmatched responses (Figure 1).

Seventy-seven participants (34.2%) could not identify even a single WHO recognized IHD symptom. The maximum score in our study was five out of seven WHO symptoms. Following table shows the number of participants corresponding to the symptom score (Table 6).

**Table 6:** Frequency of participants corresponding to the symptom score

Symptom Score	Frequency	Percentage (%)
0	77	34.2 %
1	53	23.6 %
2	64	28.4 %
3	20	8.9 %
4	9	4.0 %
5	2	0.9 %

## Discussion

This study was carried out in the Holy Family Hospital, Rawalpindi, one of the three allied hospitals of Rawalpindi Medical University. It is one of the largest public-sector hospitals in the Rawalpindi District serving a large population from all over the district including contiguous areas of Kashmir and Khyber Pakhtunkhwa.

The mean score of knowledge about IHD symptoms among the study population was 1.28 ± 1.18 while Whitaker et al. showed a mean score of 2.2 ± 1.28 for the population of Birmingham, United Kingdom (UK) [14]. This difference could be due to the difference in literacy rates and standards of living. The maximum score obtained in our study was five out of seven, which was recorded from only two participants. Chest pain (42%), pain in the arm/back (23%), and diaphoresis (19%) were the most frequently identified symptoms, which is in accordance with the findings of Zeb et al. (chest pain - 71%, pain in the arm - 14%, diaphoresis - 5%) conducted in Ayub Medical Complex, Abbottabad, Pakistan [15]. While a similar study conducted in Birmingham, UK by Whitaker et al. found chest pain (76%), pain in the arm (40%), and shortness of breath (35%) as the most frequently identified symptoms. In our study, 34% of the participants identified none of the typical IHD symptoms, while the study carried out by Whitaker et al. showed only 9% of such participants [14].

It was anticipated that the elderly population would have had a better understanding of the symptoms as compared to the younger population due to their years of life experience. However, the knowledge difference across age and gender was insignificant. These findings are in accordance with the ones reported by Goff et al., Whitaker et al., and Zhang et al. [10, 14, 16].

It was further noted that the participants of different professions showed a significant difference in knowledge about IHD symptoms. The population belonging to the professional occupation had the highest mean score of 2.00. This might be due to the difference in the educational status of the individuals belonging to different occupational groups. An interesting finding against this trend was the better score of the unemployed population compared to the unskilled and even semi-skilled population probably because of the students (5.1%) included in this category.

Formal education seemed to significantly improve the knowledge of participants regarding IHD. The highest mean score (1.94) was recorded from individuals having university-level education, whereas the lowest mean score (0.75) was that of individuals having no formal education. Though there are individual differences among the groups, the overall trend suggests increased knowledge with a higher level of education, as suggested by Zhang et al. [16]. Perhaps the reason is that formal education inculcates knowledge through scientific evidence supporting this trend.

It is generally accepted that self-experience enhances one's knowledge; however, this was negated by the current study results. A higher mean score of IHD sufferers (2.20) was found to be statistically insignificant as compared to the non-sufferers (1.26). One possible reason could be a lack of comparable size of the two groups. Individuals having relatives suffering from IHD showed a better mean score (1.73) as compared to those who had no relatives suffering from IHD (1.02). This comparison was found to be statistically significant. This result was most probably obtained because individuals are generally more caring about their near and dear ones and so are more perceptive, thus showing a better score than individuals having no such experience.

Out of the total of 27 responses recorded, 20 were irrelevant to IHD e.g., headache (4%), paralysis of the body (4%), bent hands and feet (2%), and foam at the mouth (1%). While the study conducted by Whitaker et al. shows that out of the 30 responses recorded in their study, 23 were irrelevant to IHD - collapse (9%), anxiety (7.8%), and unable to speak or swallow (6.8%) were the most frequently identified irrelevant symptoms noted by the Birmingham population [14].

However, this study is not free of limitations. Randomized sampling could not be employed. There is also a possibility of recall bias on the part of participants regarding their symptom knowledge. Thus, to give a better picture, further studies need to be done at a larger scale.

## Conclusions

It is concluded from the study that there is a paucity of knowledge about IHD symptoms among the study population. The knowledge did not seem to vary across age, gender, and socio-economic status. IHD experience among the relatives and educational status seemed to improve the knowledge of the participants. This heralds an utmost need for awareness campaigns to educate the masses to decrease pre-hospital delay and ensure timely access to healthcare.
